# Prognostic significance of inflammatory markers LMR, PLR, MPV, FIB in intermediate-and high-risk papillary thyroid carcinoma

**DOI:** 10.3389/fendo.2022.984157

**Published:** 2022-08-18

**Authors:** Canxiao Li, Jingting Li, Shijie Li, Yishen Zhao, Guandong Liu, Rui Du, Gianlorenzo Dionigi, Nan Liang, Hui Sun

**Affiliations:** ^1^ Division of Thyroid Surgery, The China-Japan Union Hospital of Jilin University, Jilin Provincial Key Laboratory of Surgical Translational Medicine, Jilin Provincial Precision Medicine Laboratory of Molecular Biology and Translational Medicine on Differentiated Thyroid Carcinoma, Changchun, China; ^2^ Division of Surgery, Istituto Auxologico Italiano IRCCS (Istituto di Ricovero e Cura a Carattere Scientifco), Milan, Italy; ^3^ Department of Pathophysiology and Transplantation, University of Milan, Milan, Italy

**Keywords:** papillary thyroid carcinoma, inflammation index, clinicopathological characteristics, recurrence, biomarkers

## Abstract

**Background:**

Lymphocyte to monocyte ratio (LMR), platelet to lymphocyte ratio (PLR), mean platelet volume (MPV) and fibrinogen (FIB) have been identified as predictive biomarkers in several malignancies. The aim of this study was to explore the association between inflammatory index with clinicopathologic features as well as recurrence risk in intermediate-to high-risk papillary thyroid carcinoma (PTC).

**Methods:**

Retrospective evaluation of 212 patients diagnosed with intermediate-to high-risk PTC who underwent surgery at China-Japan Union Hospital between 2015 and 2016. Logistic regression and receiver operating curves (ROC) were used to explore possible risk factors.

**Results:**

LMR was predictive of capsular invasion (AUC=0.595, *P*=0.017), FIB was predictive of lymph node metastasis (LN) (AUC=0.714, *P*=0.002), MPV was predictive of largest LN size ≥1cm (AUC=0.639, *P*=0.002), PLR and MPV were predictive of recurrence (AUC=0.616, *P*=0.032; AUC=0.626, *P*=0.020). In addition, FIB ≤ 2.6 (OR=6.440, 95%CI:1.777-23.336, *P*=0.005) and capsular invasion (OR=3.773, 95%CI:1.171-12.159, *P*=0.026) were identified as independent risk factors for lymph node metastasis by multivariate analysis. In addition, LN metastasis (*P*=0.048), largest LN size ≥ 1 cm (*P*=0.032), MPV > 9.4 (*P*=0.046), and PLR ≤ 128.1 (*P*=0.032) were significantly related with recurrence. Further multivariate regression analysis revealed that PLR ≤ 128.1 was a potentially independent risk factor for recurrence. Specifically, the risk of recurrence was 2.951 times higher in patients with a PLR ≤ 128.1 compared with patients with a PLR > 128.1 (OR=2.951, 95% CI:1.238-7.037, *P*=0.015).

**Conclusion:**

In intermediate-to high-risk PTC, LMR, PLR, MPV, and FIB could predict clinicopathologic features and recurrence, with lower PLR being the potential risk factors for recurrence.

## Introduction

More and more studies show that the systemic inflammatory response has a significant impact on the clinicopathological features and prognosis of malignancies (1). Tumor-related inflammatory responses play a crucial role in the formation and development of tumors., In lung cancer, pancreatic cancer, breast cancer, and other cancers, common inflammatory indices have been shown to be safe (2–5), such as neutrophil-to-lymphocyte ratio (NLR), platelet-to-lymphocyte ratio (PLR), and lymphocyte-to-monocyte ratio (LMR). Moreover, some new inflammatory markers, such as platelet volume (MPV) and fibrinogen (FIB), have been shown not only to represent the nutritional status of patients but also to assess their prognosis (6). In general, the above inflammatory indices are practical and reliable, and they are all obtained from routine hospital examinations, which does not impose any additional burden on patients. Therefore, inflammatory index has a good application prospect as a potential tumor marker.

Thyroid cancer (TC) accounts for almost 3% of all types of cancers and is the most common endocrine malignant tumor (7). In recent decades, the incidence of thyroid cancer gradully increased. It is of vital therapeutic importance to explore the occurrence and development of thyroid cancer and to find appropriate and reliable tumor markers to reduce the burden of the disease. According to the and the 2015 American Thyroid Association (ATA) guidelines and 2012 Chinese guidelines (8, 9), the clinicopathological features of intermediate-and high-risk DTC are worse compared with low-risk DTC, and the prognosis is poorer. Therefore, it is of great importance to search for tumor markers that can predict the clinicopathological features and prognosis of intermediate-to high-risk DTC to optimize the surgical plan and further improve the prognosis of DTC.

In this study, we aim to evaluate the diagnostic value of various inflammatory indices and to analyze the correlation between the inflammatory indices and the prognosis of intermediate-high risk PTC patients, further providing the basis for individualized and accurate diagnosis and treatment of intermediate-high risk PTC.

## Materials and methods

### Eligibility

The inclusion criteria were 1) sequential enrollment and an initial diagnosis of primary PTC, and 2) patients at intermediate and high risk of recurrence as determined by histological examination and imaging.

The exclusion criteria were as followed: patients who had obtained prior anti-tumor therapy, such as chemotherapy, radiotherapy, and immunotherapy; patients diagnosed with other primary cancers, coinfections, or inflammatory disorders (excluding autoimmune thyroid diseases, such as chronic lymphocytic thyroiditis); and the absence of relevant information. Throughout the study period, we enrolled all patients who met the inclusion criteria. In addition, a follow-up of clinical characteristics and outcomes was performed. No interventions wereconducted during treatment.

### Patients and data collection

**Figure 1:**
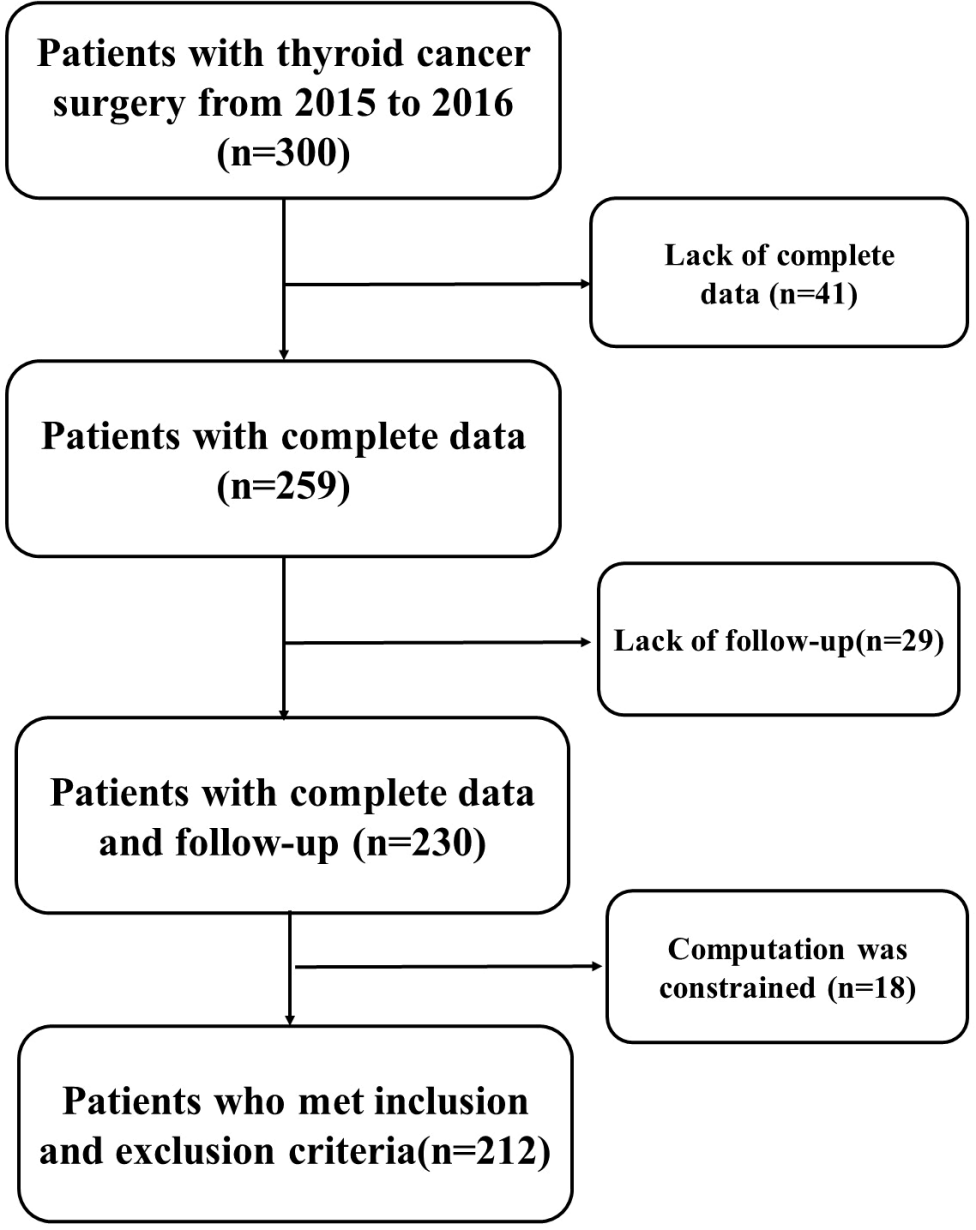
Flowchart of enrollment.

A total of 300 intermediate-high risk PTC patients were enrolled. Preoperative complete blood count, clinical and histological data, and follow-up data were available for 212 patients (**Figure 1**).

### Ethics

This study was approved by the Ethic Review Board of China-Japan Union Hospital(2021081001). Informed consent was obtained from the participants or their guardians.

### PTC diagnosis

All patients provided tumor tissue samples for histological examination or surgical excision. The diagnosis of PTC was made using the guidelines (10). Tumor size was identified as the largest tumor diameter detected on postoperative pathologic report.

### Measurement of biomarkers

Platelet-to-lymphocyte ratio (PLR) is the ratio of platelets to lymphocytes, while lymphocyte-to-monocyte ratio (LMR) is the ratio of lymphocytes to monocytes. Preoperative complete blood count was used to calculate the above inflammatory indicators. All the blood examination were completed in the same laboratory of China-Japan Union Hospital of Jilin University.

### Follow-up

By January 31, 2022, follow-up ranged from 7 to 83 months, with a median of 68 months.

### Outcomes Recorded

Patient assessment periods can be divided into one month, three months, and six months, depending on the different tumor burden of each patient. If no disease progression or adverse events occur, routine postoperative examinations are performed. Initial follow-up examinations occur every six months, and if the patient’s condition is relative stable, the interval is eventually increased to one time per year.

According to the 2015 guidelines from ATA (9), recurrence was evaluated according to both biochemical incomplete response and structural incomplete response. Biochemical incomplete response was identified as: negative imaging and suppressed Tg ≥ 1 ng/ml or stimulated Tg ≥ 10 ng/ml or rising anti-Tg antibody levels. Structural incomplete response was identified as: structural or functional evidence of disease with any Tg levels with or without anti-Tg antibodies.

### Statistical analysis

All the data analysis were conducted upon the SPSS package version 23.0 (SPSS). All data are expressed as mean, standard deviation (SD), interquartile range (IQR) and median accordingly. Categorical variables were compared and by rank sum test and Fisher’s exact test. The logistic regression models were applied to explore the risk factors of the prognosis of PTC. The threshold for statistical significance was set as *P* < 0.05. The receiver-operating characteristic curve (ROC curve) is applied to evaluate the diagnostic capabilities.

## Results

### Basic characteristic features of the patients

As illustrated in **Table 1**, a total of 212 patients were included in this study, including 57 men (26.9%) and155 women (72.1%), and a mean age of 39.3 ± 9.5 years. Postoperative pathology confirmed that 109 cases (51.4%) were reported with capsular invasion. Lymph node metastases were found in 193 (91.0%), of which 45 (21.2%) had central lymph node metastases and 148 (69.8%) with lateral neck lymph node metastases. 117 cases (48.5%) were at intermediate risk and 124 cases (51.5%) were at high risk. After a median follow-up of 68 months, 34 (16%) patients were observed to have recurrence.

**Table 1:** Baseline clinicopathological features of PTC patients.

Features	N (%)	Features	N (%)
**Total**	212	**Hashimoto’s thyroiditis**	
**Sex**		No	160 (75.5%)
Female	155 (73.1%)	Yes	52 (24.5%)
Male	57 (26.9%)	**Recurrence risk**	
**Age (years)**	39.3 ± 9.5	intermediate risk	106 (50%)
**Largest lymph nodes size (cm)**	0.5 (0.3-1.2)	high risk	106 (50%)
**Multifocality**		**Type of operation**	
No	102 (48.1%)	Lobectomy thyroidectomy+ CND	5 (2.4%)
Yes	110 (51.9%)	Subtotal thyroidectomy+ CND	2 (0.9%)
**Bilateral**		Subtotal thyroidectomy+ CND+ Ipsilateral LND	3 (1.4%)
No	127 (59.9%)	Subtotal thyroidectomy+ CND+ Bilateral LND	2 (0.9%)
Yes	85 (40.1%)	Total thyroidectomy+ CND	42 (19.8%)
**T stage**		Total thyroidectomy+ CND+ Ipsilateral LND	119 (56.2%)
T1+T2	103 (48.6%)	Total thyroidectomy+ CND+ Bilateral LND	39 (18.4%)
T3+T4	109 (51.4%)	**Inflammation index**	
**N stage**		LMR	5 (4.1-6.2)
N0	19 (9%)	PLR	121.9 (99.3-152.7)
N1a	45 (21.2%)	MPV	9 (8.3-9.8)
N1b	148 (69.8%)	FIB	2.5 (2.3-2.9)
**TNM stage**		**Recurrence**	
I+II	210 (99.1%)	No	178 (84%)
III+IV	2 (0.9%)	Yes	34 (16%)
**Capsule invasion**		**Follow up time (months)**	68 (7-83)
No	103 (48.6%)		
Yes	109 (51.4%)		

CND, central neck dissection; LND, lateral neck dissection; LMR, lymphocyte-to-monocyte ratio; PLR, platelet-to-lymphocyte ratio; MPV, average platelet volume; FIB, fibrinogen.

### Predictive performance of inflammatory indices

**Figure 2:**
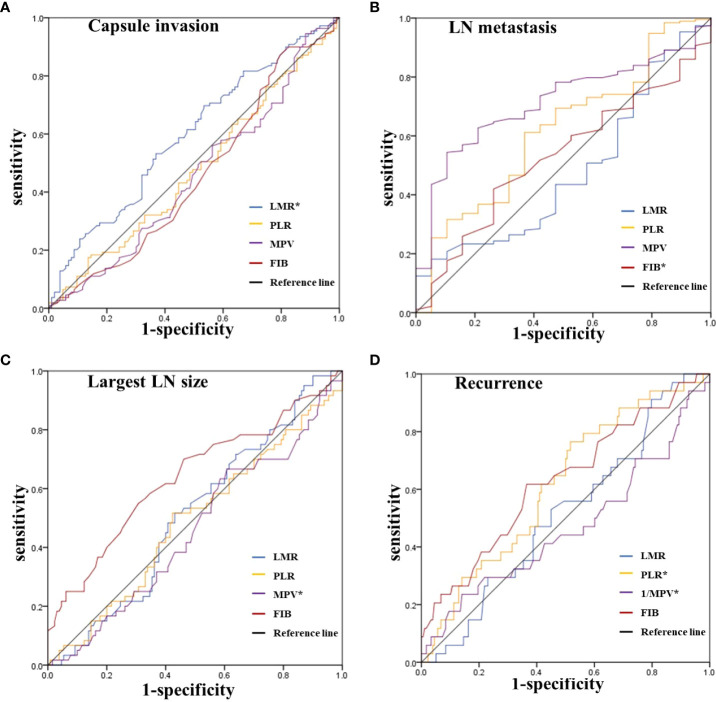
ROC curves for the preoperative LMR, PLR, MPV and FIB to predict **(A)** Capsule invasion, **(B)** LN metastasis, **(C)** Largest LN size, and **(D)** Recurrence in intermediate-high risk PTC patients. *Statistically significant *P-values* (*P* < 0.05). LMR, lymphocyte-to-monocyte ratio; PLR, platelet-to-lymphocyte ratio; MPV, average platelet volume; FIB, fibrinogen; LN, lymph node; PTC, Papillary thyroid carcinoma.

As expressed in **Figure 2**, we further evaluated the predictive performance of each inflammatory index for capsular invasion, lymph node metastasis (LN), largest LN size, and recurrence using the ROC curve. The respective AUC values are shown in **Table 2**. First, LMR was found to have significant discriminatory power for predicting capsular invasion (AUC=0.595, *P*=0.017). However, PLR, MPV, and FIB had more modest discriminatory power. The predictive value of each inflammatory index in LN metastases and the largest LN size was then assessed, and we discovered that patients with a lower FIB were more likely to have lymph node metastases (*P*=0.002), and when the cut-off value of FIB was 2.6, the AUC was 0.714, the specificity was 0.895, and the sensitivity was 0.544. Further analysis showed that the largest LN size in patients with a higher MPV (AUC=0.639, *P*=0.002) was closer to 1cm. Finally, we examined how well inflammatory indices predicted recurrence. We found that both PLR and MPV were significantly predictive of recurrence, with AUC values of 0.616 (*P*=0.032) for PLR and 0.626 (*P*=0.020) for MPV, respectively. As for PLR, the cut-off was 128.1, the specificity and sensitivity were 48.3% and 51.7%, respectively. As for MPV, the cut-off value was 9.4, the specificity was 61.8% while sensitivity was 63.5%.

**Table 2:** The summary of area under the ROC curve.

Features	AUC	95% Cl	*P*-value	Cut-off	sensitivity	specificity
**Capsule invasion**						
LMR	0.595	0.518-0.671	0.017	4.9	0.532	0.631
PLR	0.477	0.399-0.555	0.569	/	/	/
MPV	0.457	0.379-0.535	0.279	/	/	/
FIB	0.456	0.378-0.534	0.272	/	/	/
**LN metastasis**						
LMR	0.478	0.346-0.610	0.754	/	/	/
PLR	0.614	0.484-0.745	0.100	/	/	/
MPV	0.527	0.406-0.649	0.694	/	/	/
FIB	0.714	0.617-0.810	0.002	2.6	0.544	0.895
**Largest LN size≥1cm**						
LMR	0.510	0.425-0.595	0.826	/	/	/
PLR	0.488	0.399-0.577	0.794	/	/	/
MPV	0.639	0.550-0.728	0.002	9.4	0.550	0.692
FIB	0.457	0.370-0.545	0.345	/	/	/
**Recurrence**						
LMR	0.514	0.415-0.613	0.791	/	/	/
PLR	0.616	0.519-0.714	0.032	128.1	0.517	0.483
MPV	0.626	0.520-0.733	0.020	9.4	0.618	0.635
FIB	0.467	0.350-0.583	0.538	/	/	/

LMR, lymphocyte-to-monocyte ratio; PLR, platelet-to-lymphocyte ratio; MPV, average platelet volume; FIB, fibrinogen.

For P ≤0.05.

### Lower FIB and capsular invasion as risk factors for lymph node metastasis

To further discuss the risk factors for LN metastasis, the univariate and multivariate regression were applied(**Table 3**). According to univariate analysis, FIB ≤ 2.6 (*P*=0.001), age ≤ 55 (*P*=0.036), and capsular invasion (*P*=0.012) were significantly associated with lymph node metastasis. Further multivariate analysis revealed that FIB ≤ 2.6 and capsular invasion were potentially independent risk factors for lymph node metastasis. Specifically, the risk of lateral LN metastasis was increased 6.440-fold in patients with FIB ≤ 2.6 compared with patients with FIB > 2.6 (OR =6.440, 95% CI: 1.777-23.336, *P*=0.005). Patients with capsular invasion had a 3.773-fold higher risk of lateral LN metastasis than those without capsular invasion (OR =3.773, 95%CI: 1.171-12.159, *P*=0.026).

**Table 3:** Univariate and multivariate regression analyses of the possible risk factors for LN metastasis.

Features	N0	N1			Univariate *p*-value	Multivariate	
			χ^2^ value	Cramer’s V		OR (95%CI)	*p-*value
**Age (year)**			6.842	0.180	0.036		
≤55	16	187					
>55	3	6					
**Sex**			2.842	0.116	0.092		
female	17	138					
male	2	55					
**Bilateral**			0.630	0.055	0.427		
No	13	114					
Yes	6	79					
**Multifocality**			1.892	0.094	0.169		
No	12	90					
Yes	7	103					
**Capsule invasion**			6.333	0.173	0.012		0.026
No	4	99				1	
Yes	15	94				3.773 (1.171-12.159)	
**Coexisting thyroiditis**			0.036	0.013	0.786		
No	14	146					
Yes	5	47					
**Largest tumor size (cm)**			0.000	0.001	0.991		
≤1	8	81					
>1	11	112					
**FIB**			11.799	0.236	0.001		
FIB ≤ 2.6	3	110				6.440 (1.777-23.336)	0.005
FIB>2.6	16	83				1	

### Lower PLR was a potential risk factor for recurrence

Similarly, we analyzed the possible risk factors for recurrence (**Table 4**). According to univariate analysis, LN metastases (*P*=0.048), largest LN size ≥ 1cm (*P*=0.032), MPV > 9.4 (*P*=0.046), and PLR ≤ 128.1(*P*=0.032) were significantly related with recurrence. The multivariate analysis further suggested that PLR ≤ 128.1 was a potentially independent risk factor for recurrence. Specifically, the risk of recurrence was 2.951 times higher in patients with a PLR ≤ 128.1 than in those with a PLR > 128.1 (OR =2.951, 95% CI: 1.238-7.037, *P*=0.015).

**Table 4:** Univariate and multivariate regression analyses of the possible risk factors for recurrence.

Features	No recurrence group	Recurrence group	χ^2^ value	Cramer’s V	Univariate *p*-value	Multivariate	
						OR (95%CI)	* P*-value
**Age (year)**			0.169	0.028	>0.05		
≤55	170	33					
>55	8	1					
**Sex**			0.131	0.025	0.717		
female	131	24					
male	47	10					
**Largest tumor size (cm)**			0.429	0.045	0.513		
≤1	73	16					
>1	105	18					
**Bilateral**			0.020	0.010	0.888		
No	107	20					
Yes	71	14					
**Multifocality**			0.781	0.061	0.377		
No	88	14					
Yes	90	20					
**Capsule invasion**			0.863	0.064	0.353		
No	84	19					
Yes	94	15					
**Coexisting thyroiditis**			0.340	0.040	0.560		
No	133	27					
Yes	45	7					
**T stage**			0.863	0.064	0.353		
I+II	84	19					
III+IV	94	15					
**N stage**			3.986	0.137	0.048		
N0	19	0					
N1	159	34					
**Largest LN size(cm)**			4.593	0.155	0.032		
≤1	112	18					
>1	44	16					
**TNM stage**			1.729	0.090	0.296		
I+II	177	33					
III+IV	1	1					
**PLR**			7.106	0.183	0.008		
>128.1	92	26				1	
≤128.1	86	8				2.951 (1.238-7.037)	0.015
**MPV**			3.985	0.137	0.046		
>9.4	116	16					
≤9.4	62	18					

## Discussion

In the tumor microenvironment, the inflammatory response is a double-edged sword that contributes to both the progression and suppression of tumor growth (11). In recent years, inflammatory index has been identified as a new tumor marker based on host inflammatory response (12). According to studies, there is a relationship between inflammatory index, clinicopathological features as well as prognosis in several cancers, such as pancreatic cancer, lung cancer, and thyroid cancer (13–15). The incidence of thyroid cancer is increasing annually, but there are few studies on the association between thyroid tumors and inflammatory index. Therefore, this study aims to clarify and evaluate the application potential of inflammatory index as a PTC tumor marker. Compared with other studies, this study is aimed to explore the association between inflammatory index (LMR, PLR, MPV, and FIB) and recurrence risk in intermediate-to high-risk PTC.

Several previous studies have suggested the association between invasion and inflammatory index, however the conclusions inconsistent (16–18). In this study, LMR was found to be predictive of capsular invasion for the first time (AUC=0.595, *P*=0.017). The exact mechanism is currently unclear. Possible reasons include: circulating monocytes may develop into tumor-associated macrophages (TAMs) and myeloid-derived suppressor cells (MDSCs) (19, 20). They contribute to tumor cell growth, invasion, and metastasis by accelerating the transformation between epithelium and stroma, while lymphocytes could promote cytotoxins production, such as perforin, and the release of various inflammatory mediators that directly or indirectly play antitumor roles (21, 22). Therefore, LMR may reflect the state of the host immune system to some extent and is likely to be a tumor marker in PTC patients.

Patients often have impaired coagulation and fibrinolysis systems, and FIB is the highest coagulation factor in plasma. Therefore, it is worthwhile to investigate the potential of FIB as a tumor marker in PTC patients. Our study suggests that FIB is predictive of LN metastases (AUC=0.714, *P*=0.002), and further multivariate analysis showed that FIB ≤ 2.6 (OR =6.440, 95%CI: 1.777-23.336, *P*=0.005) is an independent risk factor for LN metastases. Similarly, FIB (23–25)also shows a good application prospect in other tumors such as breast cancer, renal cancer, urothelial carcinoma, etc.

In this study, ROC analysis revealed a significant association between PLR and recurrence, and further multivariate regression analysis implied that PLR ≤ 128.1 was a possible independent risk factor for recurrence. Notably, the risk of recurrence was 2.951-fold higher compared with patients with PLR ≤ 128.1 than that of patients with PLR > 128.1 (OR =2.951, 95%CI:1.238-7.037, *P*=0.015). This may be due to the fact that platelets may be increased by the release of numerous inflammatory mediators. Activated platelets can release platelet-activating factor (PAF), platelet-derived growth factor (PDGF), and vascular endothelial growth factor (VEGF), as well as other cytokines, which could enhance tumor-induced blood vessel development and promote extracellular matrix degradation, increasing tumor growth and distant metastasis (26).

However, in recent years, it is reported that platelet volume is more closely related to platelet activation compared with platelet number (27). The MPV, i.e., average platelet volume, provides information about the average platelet size, which indicates the platelet generation rate and stimulation (28). According to a number of studies, larger platelets seems to have higher metabolic and enzymatic activity than smaller ones (29). Therefore, more and more attention has been paid to the critical role of MPV in tumor evaluation. Osada (30) found that the MPV of gastric cancer patients was higher than that of 20 healthy control subjects. Similarly, we also discovered that higher MPV was predictive of the largest LN size ≥ 1cm (AUC=0.639, *P*=0.002).

This study has several limitations. First, this was a retrospective analysis. Second, though we had excluded patients with diseases that could affect complete blood count, there still might be some other possible affective factors undetectable or predictable. Finally, this study is based on one single center data, and the sample size might be limited, so it is necessary to conduct a large-scale prospective study to further clarify the scope and mechanism.

## Conclusion

In summary, our study preliminarily investigated the potential application of inflammatory indices (LMR, PLR, MPV, and FIB) in moderate-to high-risk PTC. Specifically, LMR was predictive of capsular invasion, lower FIB could predict the possibility of lymph node metastasis, lower MPV was predictive of largest LN size ≥ 1 cm, and PLR was predictive of recurrence. Remarkably, the risk of recurrence was higher in patients with lower PLR (≤128.1). This suggests that LMR, PLR, MPV, and FIB may be possible potential biomarkers for evaluating the clinicopathologic features as well as prognosis of intermediate-and high-risk PTC.

## Data availability statement

The raw data supporting the conclusions of this article will be made available by the authors, without undue reservation.

## Ethics statement

The studies involving human participants were reviewed and approved by the China-Japan Union Hospital Institutional Review Board. The patients/participants provided their written informed consent to participate in this study. Written informed consent was obtained from the individual(s) for the publication of any potentially identifiable images or data included in this article.

## Author contributions

HS, NL, CL and JL contributed to the study design and conception. CL, SL, YZ, GL, RD contributed to the data collection and analysis. All authors proposed many professional suggestions when data analysis. The first draft of the manuscript was completed by CL and JL. NL and GD contributed much effort to manuscript reviewing and editing. HS was responsible for study supervision. CL and JL contributed equally to this study and shared first authorship. All authors approved for the publication of manuscript.

## Funding

This study was sponsored by National Nature Science Foundation of China [81972499]; the Jilin Province Science and Technology Development Project [20210402011GH; 20210101342JC]; the Project of Jilin Provincial Finance Department [2020SCZ03; 2021SCZ23]; Jilin University Bethune Project [2020B14].

## Conflict of interest

The authors declare that the research was conducted in the absence of any commercial or financial relationships that could be construed as a potential conflict of interest.

## Publisher’s note

All claims expressed in this article are solely those of the authors and do not necessarily represent those of their affiliated organizations, or those of the publisher, the editors and the reviewers. Any product that may be evaluated in this article, or claim that may be made by its manufacturer, is not guaranteed or endorsed by the publisher.
